# The Predictive Performance of Depressive Symptoms in the Second Trimester of Gestation for Postnatal Depressive Symptoms in a Primary Care Setting

**DOI:** 10.1089/whr.2021.0024

**Published:** 2021-10-04

**Authors:** Kwong Ho Tam, In Wong

**Affiliations:** ^1^ Ocean Gardens Health Centre, Health Bereau, Macau SAR, China.; ^2^ Praia do Manduco Health Centre, Health Bureau, Macau SAR, China.

**Keywords:** predictive performance, second trimester, postnatal depressive symptoms, Macau

## Abstract

***Purpose:*** The aim of this study was to determine and quantify the predictive performance of depressive symptoms in the second trimester for postnatal depressive symptoms and to raise awareness of psychosocial problems.

***Methods:*** This was a prospective longitudinal study. The experimental group comprised Chinese women who completed the Edinburgh Postnatal Depression Scale (EPDS) two times (once during the second trimester and once at 6–8 weeks postdelivery) at Fong Son Tong Health Center (CSSL), Macau. Descriptive statistics were used to analyze the collected data. The predictive performance was determined using paired *t*-tests and receiver operating characteristic curves. The control group was a pre-existing group that had only completed the EPDS at 6–8 weeks postdelivery. Finally, postnatal depressive symptoms (EPDS score ≥12) were compared between the experimental and control groups.

***Results:*** In the experimental group, a total of 160 women completed the EPDS during the second trimester, and 137 (85.6%) completed the EPDS at 6–8 weeks postdelivery. The EPDS score in the second trimester was positively correlated with the score at 6–8 weeks postdelivery. The optimal threshold value of the EPDS score in the second trimester is ≥7. Compared with the control group, there was statistical significance in the detection of postnatal depressive symptoms in the experimental group.

***Conclusions:*** Screening for possible depressive symptoms in the second trimester and actively following up with at-risk women in the postnatal period may be an effective strategy for improving postnatal mental health outcomes.

## Introduction

Although delivering a baby is typically a happy event, many women develop depressive symptoms and disorders after delivery.^1^ Some women will present with postnatal depressive symptoms, and these women are more likely to develop postnatal depression than women who do not present with such symptoms.^2–3^ Postnatal depression refers to minor and major depressive episodes within the first 12 months after delivery,^4^ and most often within the first few months after childbirth.^5^ The estimated prevalence of postnatal depression varies widely among different studies, ranging from ∼6% to 16%.^5–7^ Some women with postpartum major depression may experience suicidal ideation or obsessive thoughts of harming their infants, but they often do not actively seek help.^8^ However, antenatal depressive symptoms are an important risk factor for postnatal depression, with a relative risk of 5.6.^7^ The 10-question Edinburgh Postnatal Depression Scale (EPDS) is one of the screening tools for perinatal depression.^7^ The clinical and epidemiological value of the EPDS has been confirmed by several validation studies. In the Chinese version of the EPDS, using a cutoff point of 9/10 for detecting depression in Chinese women at 6 weeks postpartum had a sensitivity of 82% and a specificity of 86%; a cutoff point of 12/13 had a sensitivity of 41% and a specificity of 95%.^9^

The Macau Health Bureau comprises one general hospital and eight health centers. The Fong Son Tong Health Center (CSSL) is a primary care setting in Macau, and most patients are Macau citizens and Chinese. Perinatal care is free to all Macau citizens. Most pregnant women receive regular antenatal care in health centers and are voluntarily screened for postnatal depressive symptoms with the EPDS at 6–8 weeks postdelivery. If the EPDS score is ≥12, further evaluation is provided, and referrals to a psychologist, psychiatrist, social worker, or the emergency room are made if necessary. According to the Macau Health Survey,^10^ from 2013 to 2016, 87.2%–90.8% of pregnant women received antenatal care in health centers, and 15.7%–19.3% had maternal postnatal checkups. For the early identification of and intervention for postnatal depression, screening in the antenatal period and regular active follow-ups may be an appropriate method.

The aim of this study was to determine and quantify the predictive performance of depressive symptoms in the second trimester for postnatal depressive symptoms. Finally, this study raises awareness of psychosocial problems and suggests improvements for postnatal mental health.

## Materials and Methods

This was a prospective longitudinal study conducted in a primary care setting (CSSL, Macau). The inclusion criteria included pregnant women who (1) received antenatal care in the CSSL, (2) had no medical history of psychiatric problems, and (3) were Chinese. The experimental group data was collected from October 2018 to April 2019. The control group included pre-existing data from October 2017 to April 2018.

In the experimental group, all eligible women were systematically selected and invited to voluntarily participate in the study, and informed consent was obtained. Participants were asked to complete the 10-item EPDS (Chinese version) twice. The EPDS was first completed during the second trimester of gestation, while undergoing the O'Sullivan test (24–28 weeks of gestation), which was guided by a trained nurse; the EPDS was completed again at 6–8 weeks postdelivery during maternal postnatal checkups. If pregnant women did not make appointments for maternal postnatal checkups, they were contacted through telephone or the questionnaire was sent through e-mail. If women received an EPDS score ≥12 in any questionnaire, we referred them to specialists or other service networks as appropriate. This study was designed to have a statistical power level of 0.8 and a significance level of 0.05. The anticipated effect size (Cohen's *d*) is 0.5. According to these parameters, it would be necessary to include ∼130 individuals.

Descriptive statistics were used for the analysis of collected data. We used an EPDS score ≥12 at 6–8 weeks postdelivery as the “endpoint” due to our guidelines. A paired *t*-test was used to compare the changes in EPDS scores over time. *p* < 0.05 was considered statistically significant. The predictive performance of the second trimester EPDS score was determined using receiver operating characteristic (ROC) curves. The sensitivity, specificity, positive predictive value (PPV), and negative predictive value (NPV) for different EPDS threshold values in the second trimester were calculated and compared to the “endpoint.” Youden's index was used as a criterion for choosing the optimal threshold for the EPDS score in the second trimester.

To confirm that the experimental group could detect more women with postnatal depressive symptoms, we collected pre-existing data as a control group that performed the current screening method that only completed the EPDS at 6–8 weeks postdelivery. Then, the percentage of EPDS scores (≥12) at 6–8 weeks postdelivery was compared between the experimental and control groups. The study was approved by the Medical Ethics Committee of Health Bureau, Macau.

## Results

**FIG. 1.**
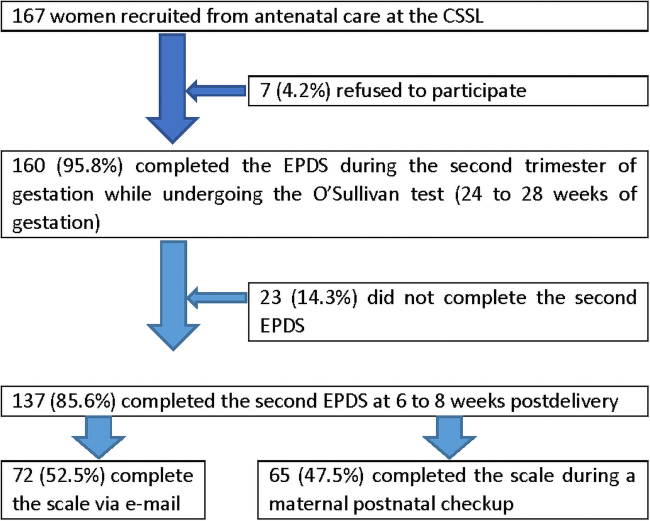
The implementation of the experimental group.

A total of 167 Chinese women were recruited for the experimental group (Fig. 1). Their ages ranged from 20 to 42 years, with a mean age of 30.5 years. A total of 160 women completed the first EPDS in the second trimester. Then, 137 (85.6%) women completed the EPDS at 6–8 weeks postdelivery, including 72 (52.5%) women who completed it through e-mail and 65 (47.5%) who completed it during a maternal postnatal checkup.

**FIG. 2.**
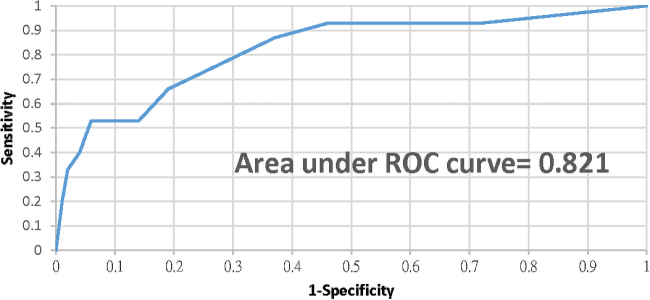
Receiver operator characteristic curve for the EPDS score at 6–8 weeks postdelivery using the EPDS score in the second trimester. EPDS, Edinburgh Postnatal Depression Scale.

In the experimental group, there was no statistical significance in the mean EPDS scores between the second trimester (6.31, standard deviation [SD] = 4.04) and at 6–8 weeks postdelivery (6.59, SD = 4.61), and the *p*-value was 0.84. The paired *t*-test showed no statistical significance in the EPDS scores between the second trimester and 6–8 weeks postdelivery (*p*-value = 0.73). Table 1 shows the sensitivity, specificity, PPV, and NPV for different EPDS threshold values in the second trimester compared to the EPDS score (≥12) at 6–8 weeks postdelivery. Generally, the sensitivity decreased progressively as the threshold value increased, while the specificity increased progressively as the threshold value decreased. The area under the ROC curve (Fig. 2) indicates an accuracy of 82% (confidence interval 95%: 78%–85%) for using the second trimester EPDS scores to predict postnatal depression. After using Youden's index, the optimal threshold value for EPDS in the second trimester was ≥7; 42.5% of cases met this threshold, with a sensitivity and specificity of 86.7% and 63.4%, respectively.

**Table 1.** Sensitivity, Specificity, Positive Predictive Value, and Negative Predictive Value for Each Edinburgh Postnatal Depression Scale (EPDS) Cutoff Point in the Second Trimester Compared to the EPDS Score (≥12) at 6–8 Weeks Postdelivery^a^ and Youden's Index

Threshold value	*n* (%)	Sensitivity (%)	Specificity (%)	PPV (%)	NPV (%)	Youden's index
≥4	68 (49.6)	93.3	27.7	14.7	96.9	0.210
≥5	53 (38.7)	93.3	42.9	17.9	98.0	0.362
≥6	38 (27.7)	93.3	53.6	21.2	98.4	0.469
≥7	26 (19.0)	86.7	63.4	24.1	97.3	0.501
≥8	21 (15.3)	73.3	75.0	28.2	95.5	0.483
≥9	16 (11.7)	66.6	81.3	32.3	94.8	0.479
≥10	9 (6.6)	53.3	86.6	34.8	93.3	0.399
≥11	9 (6.6)	53.3	92.0	47.1	93.6	0.453
≥12	7 (5.1)	53.3	93.8	53.3	93.8	0.471
≥13	3 (2.2)	46.7	95.5	58.3	93.0	0.422
≥14	2 (1.5)	40.0	96.4	60.0	92.3	0.364
≥15	1 (0.7)	33.3	98.2	71.4	91.7	0.315
≥16	1 (0.7)	20.0	99.1	75.0	90.2	0.191

^a^ Based on our practice guidelines from the Macau Health Bureau, we used an EPDS score ≥12 at 6–8 weeks postdelivery as the “endpoint” in this study.

NPV, negative predictive value; PPV, positive predictive value.

The percentage of EPDS scores (≥12) at 6–8 weeks postdelivery was 9.1% (14/167) in the experimental group and 3.2% (5/159) in the control group. Their differences were statistically significant, and the *p*-value was 0.043. This showed that screening in the second trimester can detect more women who have postnatal depressive symptoms.

## Discussion

There are well-documented risk factors for postpartum depression,^7,11–14^ and women with depressive symptoms rarely seek help, despite having access to free treatment. One study showed that a barrier to help-seeking behavior was women's inability to disclose their feelings, which was often reinforced by family members' and health professionals' reluctance to respond to the mothers' emotional and practical needs.^8^ If we can detect the risk of depressive symptoms earlier^15^ and provide interventions^16–18^ such as regularly screening for depressive symptoms during antenatal care and close observation, developing therapeutic patient-provider relationships^19^ and cooperating with other service networks if necessary can decrease the prevalence of postnatal depressive symptoms.

The EPDS was chosen for this study because of its validity and ease of use. Our nurses were well trained in using the scale, and it did not measure somatic symptoms related to women who take care of babies, such as sleeping problems.

Our data revealed that depressive symptoms in the second trimester are a suitable predictor of postnatal depressive symptoms. This finding is supported by other studies,^7,11–12,20^ which have reported that depressive symptoms in the antenatal period are positively correlated with depressive symptoms in the postnatal period in Chinese women.^13–14^ This association may be related to the added stress of pregnancy, including caring for a newborn, constant demands, and lifestyle changes. Returning to work early after birth can also lead to perinatal depression.^21^ A study showed that depression in the second trimester is also common due to the transition to parenthood.^22^ In conclusion, the evaluation of whether a pregnant woman has antenatal depressive symptoms appears to predict postnatal depressive symptoms. Postnatal depressive symptoms may be caused by prenatal factors. Routine screening during the second trimester could enhance detection and provide important information regarding interventions for postnatal depressive symptoms.

To quantify the predictive performance of depressive symptoms in the second trimester for postnatal depressive symptoms, the best threshold value of the EPDS score in the second trimester was ≥7. This is lower than the threshold value recommended for perinatal depression screening. This difference might be due to Chinese women showing the traditional trait of self-restraint^23^ or dynamic changes in depressive symptoms during the perinatal period. Another possible reason was the selection of the endpoint in our study. We believe that quantifying the EPDS score in the second trimester can raise awareness among health workers of the need to examine pregnant women's psychological problems or social stressors.

Compared with only screening for postnatal depressive symptoms at 6–8 weeks postdelivery, screening both in the second trimester and at 6–8 weeks postdelivery can detect more possible cases. According to our results, 42.5% of pregnant women are at risk of experiencing mental health problems postdelivery and need to be followed up with actively. Treating postnatal depression increases the workload during a maternal postnatal checkup ∼2.5-fold based on the coverage rate reported by the 2016 Macau Health Survey. Therefore, the burden of postnatal depressive symptoms on our health system must be considered.

Although it contributes to the literature, this study has several limitations: (1) the EPDS is a screening tool that cannot accurately diagnose depression or establish its prevalence.^24^ (2) The endpoint in this study was an EPDS score ≥12 at 6–8 weeks postdelivery, which cannot represent the actual condition of postnatal depression. (3) The control group was pre-existing data using the current screening method. Although there was some bias, we manipulated the inclusion criteria as an experimental group to reduce bias. (4) Some risk factors were not considered in this study, such as basic characteristics and the outcome of delivery.

## Conclusion

To increase the awareness of postnatal depressive symptoms, it is important to pay more attention to women in the antenatal period. Screening for possible depressive symptoms in the second trimester and actively following up with women who are prone to postnatal depressive symptoms may be an effective strategy to improve postnatal mental health outcomes.

## Ethical Approval

Ethical approval was obtained from the Medical Ethics Committee of Health Bureau, Macau (no. 0679/SCSD/N/2018).

## Abbreviations Used

CSSL: Fong Son Tong Health Center
EPDS: Edinburgh Postnatal Depression Scale
NPV: negative predictive value
PPV: positive predictive value
ROC: receiver operating characteristic
SD: standard deviation
